# Mental health of general practitioners and family medicine specialists 2 years into the COVID-19 pandemic

**DOI:** 10.3389/fpubh.2025.1464639

**Published:** 2025-03-12

**Authors:** Marija Zafirovska, Jelena Danilenko, Aleksandar Zafirovski, Kristien Coteur, Heidrun Lingner, Cristián Andrés Frigolett, Milena Cojić, Mustafa Kürşat Şahin, Carmen Iliana Busneag, Nicola Buono, Aleksander Stepanović, Christine Brütting, Lyubomir Kirilov Kirov, Zaim Jatić, Liljana Ramasaco, Monika Brovč, Vanja Lazić, Erjona Abazaj, Ljubin Šukriev

**Affiliations:** ^1^Association of General Practice/Family Medicine of South-East Europe (AGP/FM SEE), Skopje, North Macedonia; ^2^Faculty of Medicine, University of Ljubljana, Ljubljana, Slovenia; ^3^Department of Family Medicine, Rīga Stradiņš University, Riga, Latvia; ^4^MFD Health Group, Riga, Latvia; ^5^General Hospital Jesenice, Jesenice, Slovenia; ^6^Clinical Institute of Radiology, University Medical Centre Ljubljana, Ljubljana, Slovenia; ^7^Department of Public Health and Primary Care, KU Leuven, Leuven, Belgium; ^8^Department of Medical Psychology OE 5430, Center for Public Health and Healthcare, Hannover Medical School, Hannover, Germany; ^9^Leuven Biostats, Biomedical Sciences, KU Leuven, Leuven, Belgium; ^10^Department of Family Medicine, Faculty of Medicine, University of Montenegro, Podgorica, Montenegro; ^11^Department of Family Medicine, School of Medicine, Ondokuz Mayıs University, Samsun, Türkiye; ^12^Department of Kinetic Therapy and Special Motricity, "Spiru Haret" University, Bucharest, Romania; ^13^Medical Department, National Romanian Television, Bucharest, Romania; ^14^Individual Medical Office of Family Medicine "Dr. Busneag Carmen", Bucharest, Romania; ^15^National Society of Medical Education in General Practice (SNAMID), Caserta, Italy; ^16^Department of Family Medicine, Faculty of Medicine, University of Ljubljana, Ljubljana, Slovenia; ^17^Primary Healthcare of Gorenjska, Public Medical Center in Škofja Loka, Škofja Loka, Slovenia; ^18^Institute for General Medicine, Faculty of Medicine, University of Halle-Wittenberg, Halle (Saale), Germany; ^19^General Medicine, Faculty of Medicine, Sofia University ‘’St. Kliment Ohridski’’, Sofia, Bulgaria; ^20^National Association of General Practitioners in Bulgaria, Sofia, Bulgaria; ^21^Public Institution Health Centre of Sarajevo Canton, Sarajevo, Bosnia and Herzegovina; ^22^Faculty of Medicine, University of Sarajevo, Sarajevo, Bosnia and Herzegovina; ^23^Nursing Department, Faculty of Technical Medical Sciences, University “Aleksander Xhuvani”, Elbasan, Albania; ^24^National Institute of Public Health (NIJZ), Ljubljana, Slovenia; ^25^Association of Teachers in General Practice/Family Medicine, Zagreb, Croatia; ^26^Health Center Zagreb - Centar, Zagreb, Croatia; ^27^European General Practice Research Network, Zagreb, Croatia; ^28^Department of National Reference Laboratories, Institute of Public Health, Tirana, Albania

**Keywords:** general practice, family practice, mental health, COVID-19, anxiety, depression, fear of COVID-19

## Abstract

**Introduction:**

The COVID-19 pandemic has significantly impacted general medical practice by altering work structures and increasing teamwork while also adversely affecting the mental health of general practitioners and family medicine specialists. This study assesses depression, anxiety, and fear levels among general practitioners and family medicine specialists in Europe 2 years after the COVID-19 pandemic’s onset, and it explores influencing factors.

**Methods:**

This observational cross-sectional study included participants from 13 European countries. Data was collected from May to August 2022 with an anonymous online survey incorporating validated questionnaires for depression (PHQ-9), anxiety (GAD-7), and fear of COVID-19 (FCV-19S). Data analysis involved descriptive statistics, correlation tests, and linear regression.

**Results:**

A total of 1,723 participants completed the survey. Findings indicated an overall mild to moderate levels of anxiety (GAD-7: 5.4 ± 4.76) and depression (PHQ-9: 6.33 ± 5.43), and moderate fear of COVID-19 (FCV-19: 12.84 ± 5.29). Key determinants of anxiety, fear, and depression included country, at-risk status, patient non-compliance, and mental health history. Sex influenced anxiety and fear, losing co-workers to COVID-19 influenced anxiety and depression, while losing relatives or friends influenced fear.

**Conclusion:**

Two years into the pandemic, European general practitioners and family medicine specialists showed mild to moderate levels of anxiety, depression, and fear. Country, at-risk status, mental health history, and work-related challenges significantly affected mental health. Crucial interventions are needed to support healthcare workers during pandemics, focusing on protective measures, stable work environments, and coping strategies for anxiety and depression.

## Introduction

Epidemics and pandemics have been associated with increased rates of psychological distress, particularly among healthcare workers (HCWs), who are frequently exposed to high-risk environments and intense workloads (1). During viral epidemic outbreaks, studies have documented elevated rates of acute stress disorder, anxiety, burnout, depression, and post-traumatic stress disorder among HCWs (2). General practitioners (GPs) and family medicine specialists (FMSs) play a vital role in primary healthcare, yet research focusing on their mental health during pandemics remains comparatively limited relative to hospital-based HCWs.

The COVID-19 pandemic has placed an unprecedented strain on global healthcare systems, significantly affecting the well-being of primary care providers. While the pandemic has prompted beneficial changes such as enhanced teamwork and modified work systems, these benefits have been counterbalanced by increased workloads, psychological distress, and workplace stressors, including patient non-compliance, lack of social support, and exposure to misinformation and harassment from COVID-19 deniers (3–5). These stressors have the potential to influence workforce retention, care quality, and the long-term sustainability of health systems (6). Studies in Australia and Singapore have shown that GPs reported increased stress, burnout, and emotional exhaustion due to prolonged exposure to workplace challenges and a lack of institutional support during COVID-19 (7, 8). Cross-national comparisons suggest that differences in healthcare infrastructure and government support mechanisms have influenced the psychological resilience of primary care providers (9). However, there remains a lack of multinational data specifically examining the psychological impact of the pandemic on GPs and FMSs across different healthcare contexts in Europe.

This study aims to fill this research gap by providing a comprehensive analysis of depression, anxiety, and fear of COVID-19 among European GPs and FMSs 2 years into the pandemic. Unlike previous studies that focused primarily on hospital-based HCWs, our research provides new insights into how various demographic, professional, and personal factors influence mental health outcomes within primary care settings. By offering a cross-national perspective, this research provides valuable insights into the psychological well-being of primary care physicians and informs policy recommendations for mitigating future mental health burdens during public health crises.

## Materials and methods

### Study design and participants

This anonymous online observational cross-sectional survey was conducted across 13 European countries: Albania, Bosnia and Herzegovina, Bulgaria, Croatia, Germany, Italy, Latvia, Macedonia, Montenegro, Romania, Serbia, Slovenia, and Turkey. The target population was GPs and FMSs in the member-countries. Convenience sampling was used to recruit participants through various national organizations of GPs and/or FMSs in each country.

### Questionnaire development and primary endpoints

The initial questionnaire, based on an extensive literature review, was developed in English by the core team (MZ, JD, AZ, KC, LŠ), and piloted in February–March 2022. Following revisions, it was translated, back-translated, and adapted for 13 languages. The questionnaire included several sections, including sociodemographic questions, questions addressing professional and personal experiences during COVID-19, and scales for assessing depression, anxiety, and fear of COVID-19.

The primary endpoints included depression levels using the nine-item Patient Health Questionnaire (PHQ-9) scale, anxiety levels using the seven-item Generalized Anxiety Disorder (GAD-7) scale and the fear levels using the Fear of COVID-19 scale (FCV-19S). The PHQ-9 is a validated nine-item tool used for screening, diagnosing, monitoring and measuring the severity of depression, with a sensitivity and specificity of 88% for Major Depressive Disorder at a cut point of ≥10 (10, 11). The GAD-7 is a validated seven-item anxiety scale with sensitivity of 89% and specificity of 82% for general anxiety disorder at a cut point of ≥10 (12). The Fear of COVID-19 Scale is a validated test with seven Likert-type statements used to evaluate individuals’ fear of COVID-19. The test shows good reliability, with an internal consistency of *α* = 0.82 and acceptable test–retest reliability (ICC = 0.72) (13, 14).

Participants from Germany were exempted from the “number of registered patients” metric, due to different registration practices, and a surrogate parameter was used for workload comparison, which is the “number of patients treated quarterly.”

### Data collection and data analysis

The survey was conducted on the Qualtrics platform, which complies with GDPR regulations and ensures data security. No identifiable personal data, such as names, surnames, or addresses, were collected, guaranteeing participant anonymity. The survey was launched on the 23rd of May 2022 and closed on the 10th of August 2022. Our target sample size was a minimum of 50 participants per country. In total 1723 people participated in the survey. The statistical analysis was performed using the R Statistical software (version 4.0.5), with continuous variables reported as mean ± SD and categorical variables as percentages. All tests were two-tailed and a *p*-value <0.05 was considered statistically significant. The PHQ-9 and GAD-7 scales are commonly used with established cutoffs to categorize the severity of depression and anxiety. However, in this study, these scales were analyzed as continuous variables, with the reported means and standard deviations providing an overall indication of severity across the population.

To explore associations, four different tests were performed: (1) Pearson’s correlation for two continuous variables; (2) Spearman’s correlation for ordinal and continuous variables; (3) Point biserial correlation for dichotomous and continuous variables; (4) Kruskal Wallis test for categorical and continuous variables. The Kruskal-Wallis test is a non-parametric equivalent of ANOVA which shows that there are differences, e.g., between countries, without assessing how strong these differences are. Linear regression models examined factors influencing depression, anxiety, and fear. Covariates were selected using stepwise AIC (bi-directional) method.

## Results

### Demographics

Among the 1723 participants, the majority were female (76.09%), and the average age was 47 ± 12 years (See Appendix 1, Table 1). Most of the participants worked in urban areas (74.29%). There were 913 FMSs and 711 GPs. The mean years of experience were 17.04 ± 10.98. The mean number of registered patients per GP, across countries, was 1865.02 ± 871.5, while the number of patients treated quarterly by German GPs was 1251.25 ± 399.08. Over two thirds of the participants were in a relationship/married and cohabitating (67.09%; See Appendix 1, Table 1).

The PHQ-9 scores had a mean of 6.33 ± 5.43, with a median of 5, indicating an overall mild to moderate depression. The GAD-7 scores had a mean of 5.4 ± 4.76, with a median of 4, indicating an overall mild to moderate anxiety. The FCV-19S scores had a mean of 12.84 ± 5.29, with a median of 12, suggesting an overall moderate fear related to COVID-19. The statement “It makes me uncomfortable to think about Corona” had the highest mean score of 2.35, followed by “When I watch news and stories about Corona on social media, I become nervous or anxious” with a mean score of 2.19. The statement “I cannot sleep because I’m worrying about getting Corona” had the lowest mean score of 1.48.

### Univariate analysis

#### Demographic factors

There was a significant association between country of origin and depression (*p* < 0.0001), anxiety (*p* = 0.006) and fear of COVID-19 (*p* < 0.0001; See Appendix 1, Table 2). Bulgaria had the highest mean depression (8.37 ± 6.14) and anxiety scores (7.04 ± 5.75), while Montenegro had the highest mean fear of COVID-19 score (15.20 ± 4.74). Conversely, Serbia had the lowest mean depression (4.16 ± 3.90) and anxiety scores (3.65 ± 3.59), while Germany had the lowest mean fear scores (9.09 ± 2.66). Males showed a negative association with anxiety (*p* = 0.006), depression (*p* = 0.021), and fear (*p* < 0.0001; See Appendix 1, Table 3). Workplace area, i.e., urban, suburban, or rural, was only significantly associated with depression (*p* = 0.008; See Appendix 1, Table 2). GPs reported statistically significant lower levels of depression, anxiety, and fear compared to FMSs, although the relationship was relatively weak (GAD-7 r = −0.107; PHQ-9 r = −0.112; FCV-19S r = −0.149; *p* < 0.0001 for all measures; See Appendix 1, Table 3). A significant association was found between workplace sector, i.e., private, public, or a public-private partnership, and depression (*p* = 0.027) but not anxiety or fear of COVID-19. Finally, marital status was only significantly associated with fear of COVID-19 levels (*p* = 0.004) but there was no correlation found between living alone and fear of COVID-19, nor anxiety or depression (See Appendix 1, Tables 2, 3).

#### Professional and personal experiences during the pandemic

Closing practices during COVID-19 significantly increased depression levels, although the effect was small (*r* = 0.08; *p* = 0.002; See Appendix 1, Table 3). Coworker deaths were significantly associated with anxiety (*p* = 0.001) and fear of COVID-19 (*p* < 0.0001; See Appendix 1, Table 2). Workplace challenges like patient non-compliance, lack of guidance/protocols, reduced communication and staff shortages were significantly increasing levels of anxiety, depression and fear levels (*p* < 0.001), with small to moderate effects (*ρ* range: 0.114–0.303). More details are provided in Appendix 1, Table 3.

Participants categorized as “at-risk” (e.g., advanced cancer, severe asthma/COPD, pregnancy, age over 70) were more likely to experience depression (*r* = 0.108; *p* < 0.0001), anxiety (*r* = 0.086; *p* = 0.002) and fear of COVID-19 (*r* = 0.130; *p* < 0.0001; See Appendix 1, Table 3). Hospitalization for COVID-19 treatment was associated with anxiety symptoms (*p* = 0.014). Death of a close relative/friend due to COVID-19 was significantly associated with fear (*p* < 0.0001; See Appendix 1, Table 2). Pre-pandemic mental health disorders were significantly associated with fear of COVID-19 (*p* = 0.044). During the pandemic, these disorders were significantly associated with depression (*p* = 0.016), anxiety (*p* = 0.002), and fear levels (*p* = 0.016; See Appendix 1, Table 2). Seeking mental health consultation during the pandemic was positively correlated with higher levels of depression (*r* = 0.115; *p* < 0.0001), anxiety (*r* = 0.147; *p* < 0.0001), and fear of COVID-19 (*r* = 0.112; *p* < 0.0001; See Appendix 1, Table 3).

### Multivariate analysis models

#### Depression model

Statistically significant factors associated with depression levels included country, losing co-workers to COVID-19, work-related challenges, falling within the at-risk category, and pre-existing mental health disorders (See Appendix 1, Table 4). Being from Serbia and not experiencing any mental health disorders before and during the pandemic significantly lowered depression levels. Conversely, being from Turkey, falling within the at-risk category, and experiencing mental health disorders before and during the pandemic significantly increased depression levels. Work-related challenges such as staff shortage, patient non-compliance, lack of clear guidance/protocols, and problems with prescribing medicine and medical devices to chronic patients significantly increased depression levels.

#### Anxiety model

Statistically significant factors associated with anxiety levels included country, sex, losing co-workers to COVID-19, work-related challenges, falling within the at-risk category, and pre-existing mental health disorders (See Appendix 1, Table 5). Being male and from Serbia significantly lowered anxiety levels. Conversely, being from Germany, losing co-workers to COVID-19, falling within the at-risk category, having pre-existing mental health disorders, as well as staff shortages, patient non-compliance, and reduced methods of communication with the secondary/tertiary level of healthcare significantly increased anxiety levels.

#### Fear of COVID-19 model

Statistically significant factors associated with fear of COVID-19 levels included country, sex, work-related challenges, falling within the at-risk category, experiencing loss of relatives or friends to COVID-19, and pre-existing mental health disorders (See Appendix 1, Table 6). Males and participants from all countries except Turkey showed lower fear levels, while, falling within the “at-risk category,” patient non-compliance, and experiencing mental health disorders before and during the pandemic increased fear levels.

## Discussion

### Summary

This survey study explored the levels of depression, anxiety and fear of COVID-19 among GPs and FMSs in Europe, 2 years after the COVID-19 pandemic’s onset, and it explored influencing factors. Our findings revealed mild to moderate levels of anxiety and depression, and a moderate level of fear of COVID-19 among the European GPs and FMSs. The three models we developed uncovered various demographic, professional and personal factors influencing these assessed levels. Overall, country, falling within the at-risk category, patient non-compliance and mental health history were key determinants across anxiety, fear, and depression models. Sex was an influencing factor in the anxiety and fear models. Losing co-workers to COVID-19 influenced anxiety and depression levels, while losing relatives or friends to COVID-19 influenced only fear levels.

### Strengths and limitations

While most COVID-19 mental health research focuses broadly on HCWs, we targeted GPs and FMSs, and this gave us nuanced insights. The main strength of our study lies in its multinational scope and large sample size, enhancing the generalizability of our findings and enabling cross-cultural comparisons.

On the other hand, participants were predominantly from Central and Eastern European countries, except for Italy and Turkey. Convenience sampling may have introduced selection bias, potentially excluding individuals with higher depression, anxiety, and fear levels. As an exploratory and observational study, multiple test corrections were not applied. The variables in the multivariate models included overlapping categories (e.g., ‘anxiety’ and ‘anxiety, depression’), which may introduce redundancy and complicate interpretation. While this reflects the real-world complexity of comorbid conditions, future studies could explore alternative categorizations to enhance clarity. Our approach aimed to preserve the richness of the data and participants’ experiences during the pandemic. Furthermore, the ‘N/A’ category for sex was included in the analysis but represents fewer than 0.5% of the total sample, which may contribute to computational instabilities and affect the stability of the results. Despite these limitations, the present study provides interesting research directions for future studies, especially to further explore the impact of many categorical variables that were found to be significant such as country, workplace sector, and marital status on the mental health status of GPs and FMS.

### Comparison with existing literature

Various studies worldwide assessed depression, anxiety and fear in HCWs during COVID-19. In Italy, 28.1% of 139 GPs displayed moderate to severe depressive symptoms (mean PHQ-9 7.0 ± 4.8), while 31.7% displayed similar anxiety symptoms (mean GAD-7 7.4 ± 4.7) (15). Conversely, in Latvia, 24.8% of 864 HCWs experienced depression (PHQ-9 ≥ 10) and 17.2% had anxiety (GAD-7 ≥ 10) (16). French GPs also reported psychological issues (17). During the second wave of COVID-19, 79.4% of 1992 private practitioners, including GPs, experienced psychological distress, including burnout, depression, anxiety, and insomnia (18). The fear of COVID-19 varied; in Indonesia GPs reported moderate fear (mean FCV-19S score 9.0 ± 4.9) (19), while HCWs in Palestine and Malaysia reported higher fear levels (mean FCV-19S score 17.53 ± 5.78 and 19.1 ± 6 accordingly) (20, 21). Our study found lower mean PHQ-9 (6.33 ± 5.43), GAD-7 (5.4 ± 4.76) and FCV-19S (12.84 ± 5.29) scores compared to previous studies. A possible explanation for the differing levels of depression, anxiety and fear between the studies conducted in different time periods could be the enhanced understanding of the virus, improvement in work policies and protocols related to COVID-19, and the widespread availability and administration of COVID-19 vaccines (9).

In our univariate analysis, males experienced lower levels of anxiety, depression and fear, while in the multivariate analysis, being male was associated with lower levels of fear and anxiety only. Previous studies revealed men were more prone to depression (16), while women experience higher anxiety (16, 22). Female HCWs, non-physicians, and individuals with a history of anxiety showed higher anxiety and fear of COVID-19 levels (23). Studies in Palestine and Turkey also highlighted higher levels of these mental health issues among women, with the fear of infecting loved ones being the most common (20, 24, 25) In our study, age wasn’t significantly associated with mental health scores, however some studies showed younger age and fewer years of GP experience were associated with higher anxiety and depression levels (15, 16, 22).

Our univariate analysis showed that GPs exhibited lower levels of depression, anxiety, and fear compared to FMSs, although this was not statistically significant in the multivariate models. Previous studies have found that GPs experience more depression, anxiety, and burnout than hospital staff (16), and perceived their stress as work or COVID-19-related (18). They received less training on personal protective equipment (PPE) and COVID-19 patient care, less workplace support, while also worrying about income, infecting family, and blame from colleagues, which contributed to their poorer mental health (7).

A Greek study of 236 HCWs, including GPs, found rural work settings associated with a lower anxiety risk, although depression risk was not significantly affected by location (22). Our univariate analysis revealed a significant association between work area and depression, suggesting depression levels may differ by work setting. In Singapore, a study of 257 GPs revealed higher anxiety and depression levels in the public sector due to procedural changes, increased workload, and financial pressures (8). In a study of 411 frontline Egyptian physicians, fear levels were associated to the work department (26). Our study found a significant association between workplace sector and depression, but not anxiety or fear of COVID-19.

A study of 1,040 Chinese GPs found anxiety associated with inadequate facilities and poor sleep, but mitigated by training and proper PPE (27). Fear levels were influenced by chronic illness, exposure risk, and workplace safety (21). Longer working hours, working in high-risk areas, and having at-risk relatives increased depression and anxiety risk, while direct contact with COVID-19 patients did not significantly affect mental health (16, 19). In Pakistan among 400 HCWs, social media increased fear, while age, profession, and education influenced fear and depression, and PPE availability affected anxiety levels (28). Our analysis showed that work-related problems such as staff shortages, patient non-compliance, lack of clear guidance/protocols, problems with prescribing medicine and medical devices to chronic patients significantly increased the depression levels, with patient non-compliance also raising anxiety and fear. Falling within the at-risk category significantly increased depression, fear, and anxiety levels, while contact with COVID-19 correlated with anxiety only in the univariate analysis. Another study of 531 GPs, linked anxiety to higher negative perceptions and fear of COVID-19 (29). In our study, the most common fear was discomfort when thinking about COVID-19, followed by feeling nervous or anxious when exposed to related news or stories on social media.

These findings highlight the complex interplay of factors shaping mental health outcomes during the pandemic. The multivariate analysis revealed that being from Serbia significantly reduced depression and anxiety levels, yet no explanation for this effect was found in the data or existing literature. Conversely, higher anxiety levels in Germany and higher depression levels in Turkey may reflect distinct stressors, such as procedural changes or workload pressures specific to these healthcare systems. Differences in cultural norms, healthcare practices, or access to mental health resources could also contribute to these country-specific outcomes. Further research is needed to better understand these variations and their implications for supporting healthcare professionals.

### Implications for research and/or practice

The identified factors influencing anxiety, depression, and fear levels, including demographics, professional context, and personal history, highlight the multifaceted nature of mental health in this population. Our findings underscore the importance of tailored interventions and support mechanisms to address the unique challenges faced by GPs and FMSs. Policymakers and healthcare administrators should consider implementing strategies to mitigate work-related stressors and provide targeted mental health support. Future research should focus on longitudinal studies and comparative analyses across different healthcare systems and cultural contexts to further understand the pandemic’s global mental health impact.

## Conclusion

GPs and FMSs in Europe experienced mild to moderate anxiety and depression levels, and moderate fear of COVID-19 levels 2 years into the pandemic. Various demographic, professional, and personal factors, such as country, at-risk status, and mental health history, influenced these levels across the three models developed. Comparison with previous studies demonstrated varying levels of anxiety, depression, and fear across different countries and professions during different stages of the pandemic, suggesting fluctuations possibly influenced by evolving understanding, policies, and vaccination efforts. Sex disparities and workplace challenges, like staff shortages and patient non-compliance, also impacted mental health. In future pandemics, actions should be taken to reduce the risks and increase the protective factors, including addressing GPs’ and FMSs’ needs, providing mental health support at the individual level, and combating stigma through awareness at the community-level.

## Data Availability

The raw data supporting the conclusions of this article will be made available by the authors, without undue reservation.
